# Practice of Peritoneal Adhesions in Osteopathic Medicine: Part 2

**DOI:** 10.7759/cureus.43092

**Published:** 2023-08-07

**Authors:** Bruno Bordoni, Gregory T Girgenti, Allan R Escher

**Affiliations:** 1 Physical Medicine and Rehabilitation, Foundation Don Carlo Gnocchi, Milan, ITA; 2 Anesthesiology, H. Lee Moffitt Cancer Center and Research Institute, Tampa, USA; 3 Anesthesiology/Pain Medicine, H. Lee Moffitt Cancer Center and Research Institute, Tampa, USA

**Keywords:** manual therapy, surgery, osteopathic manipulation, osteopathy, myofascial, peritoneal adhesions, fascia

## Abstract

Peritoneal adhesions are an unwanted and frequent event following abdominal surgery, with a response rate that can reach 100%. The adhesions can be symptomatic, becoming a source of pain and discomfort for the patient, or asymptomatic, with possible chronic or acute visceral dysfunction. The article reviews what the diagnostic strategies are and discusses what could be the causes that lead to chronic pain in the presence of adhesions. The text reports the knowledge of the literature on the manual treatment of adhesions and illustrates possible symptoms that are not easily recognized by the clinician. To conclude, the article proposes osteopathic manual approaches derived from clinical experience and from what has been explained about the formation of peritoneal adhesions. Research must make further efforts to identify not only the causes triggering the formation of peritoneal neogenesis but also seek the most appropriate non-invasive treatments to help the patient.

## Introduction and background

Peritoneal adhesions are a frequent occurrence after abdominal surgery. The finding of post-laparotomy adhesions for the upper abdominal area is about 93% to 100%, while for the lower abdominal area, it is around 67% to a maximum of 93% [1]. Of patients undergoing abdominal surgery, approximately 18% will need to undergo another surgery to eliminate symptomatic adhesions [1]. One of the most important causes of all the reasons that cause small bowel obstructions is the presence of adhesions, which accounts for about 75% [1]. About 20% of people undergoing abdominal and pelvic surgery must undergo a second operation due to the presence of adhesions 10 years after the first operation [2]. The presence of adhesions increases the risk of adverse events during and after the surgical approach, increases hospitalization times, causes chronic abdominal and pelvic pain, and increases the costs to support these problems [2]. Adhesions are the peritoneal response to a lesion due to different causes, starting from the phenomenon of coagulation, such as surgery, the presence of inflammation, abdominal trauma, and the presence of infection [2]. If there is an alteration in the behavior of fibrin degradation, i.e., the latter is not degraded correctly, a peritoneal neoformation or adhesion is created, which is innervated and vascularized [2]. For more details, we invite the reader to consult the previous article [3]. In the literature, we can find some attempts to give definitions to adhesions, such as dividing them into "adhesion formation" (born in the surgical site), "de novo adhesion formation" (adhesions born independently of surgery), and "adhesion reformation" (the adhesions formed secondary to adhesiolysis) [2]. According to other authors, they can be classified as "type 1 or de novo" or "type 1A" (bonds formed in the absence of surgery) and "type 1B" (presence of bonds due to previous surgery); "type 2" with a further classification in "type 2A" (adhesions re-formed in the absence of surgery and only after adhesiolysis); and "type 2B" (adhesions re-formed following various surgical procedures) [2]. Other classifications take into consideration the formation time with respect to the surgical date: membranous adhesions (within the first three days); vascular adhesions (3-21 days); adhesive adhesions (2-28 days); scar adherence (months-years) [3]. The article reviews the most suitable tools to make a definite diagnosis of the presence of adhesions and the mechanisms from which chronic visceral pain derives. The article continues what the literature exposes about manual treatment for the treatment of these neoformations of the peritoneum and discusses the possible symptoms not only related to pain but also to disorders that are not easily identifiable and connected to adhesions. Osteopathic manipulative treatment (OMT) is a treatment modality that observes the patient as a whole (physical and mental), not only focusing on the symptom but also on the origin of the symptom. To do this, the osteopathic clinician uses and develops very fine and precise manual skills (as well as using tests and clinical instruments). We conclude with some proposals for osteopathic manual treatment, deriving from clinical experience and from the information brought to light in this and the previous article (first part) [3].

## Review

Diagnostic approach

Laparotomy is an invasive instrumental examination considered the approach of choice to verify the presence of peritoneal adhesions [4]. Another exam specifically used for the Douglas pouch area is transvaginal hydro-laparoscopy (also referred to as fertiloscopy); this examination can be performed with general or local anesthesia [4]. The transabdominal ultrasound-guided examination evaluates the visceral sliding sign, but the result can be operator-dependent, and the examination cannot always highlight the presence of adhesions [4]. Other non-invasive instrumental tests, such as magnetic resonance and computed tomography, can hardly evaluate the extent of adherence; it is much easier to highlight an obstruction in the acute phase and any severity [5]. The diagnosis of adhesions is mainly clinical, that is, based on the patient's visit, on the description of the symptoms and the distribution of pain, and on the anamnesis. Most diagnostic tests can only make one suspect an adhesion syndrome or possibly make a diagnosis of exclusion. The clinician will evaluate more carefully some local symptoms, such as the presence of abdominal pain (on palpation or simple movement of the patient), persistent bloating, not passing gas, abdominal distension, constipation, nausea and vomiting, patient temperature, possible blood parameters related to infection or inflammation, and other systemic parameters such as heart rate and blood pressure (in case of acute intestinal obstruction) [5]. A clinical sign of the possible presence of abdominal adhesions is Gilroy Bevan's triad: pain or pain relief depending on the position held by the patient, pain present in the scar area due to previous surgery, and tenderness of the scar assessed by palpation [6]. Palpation of the abdominal area in cases of adhesions will highlight an increase in tissue tension and pain (local or radiated), which pain is not always perceived by the patient; the patient does not always know the cause of his symptom. If, in the clinical history, the patient reveals a previous abdominal operation or a history of pathologies related to a systemic inflammatory status (metabolic syndrome, chronic pathologies), the clinician should always palpate the abdominal area [3].

Chronic pain

**Figure 1:**
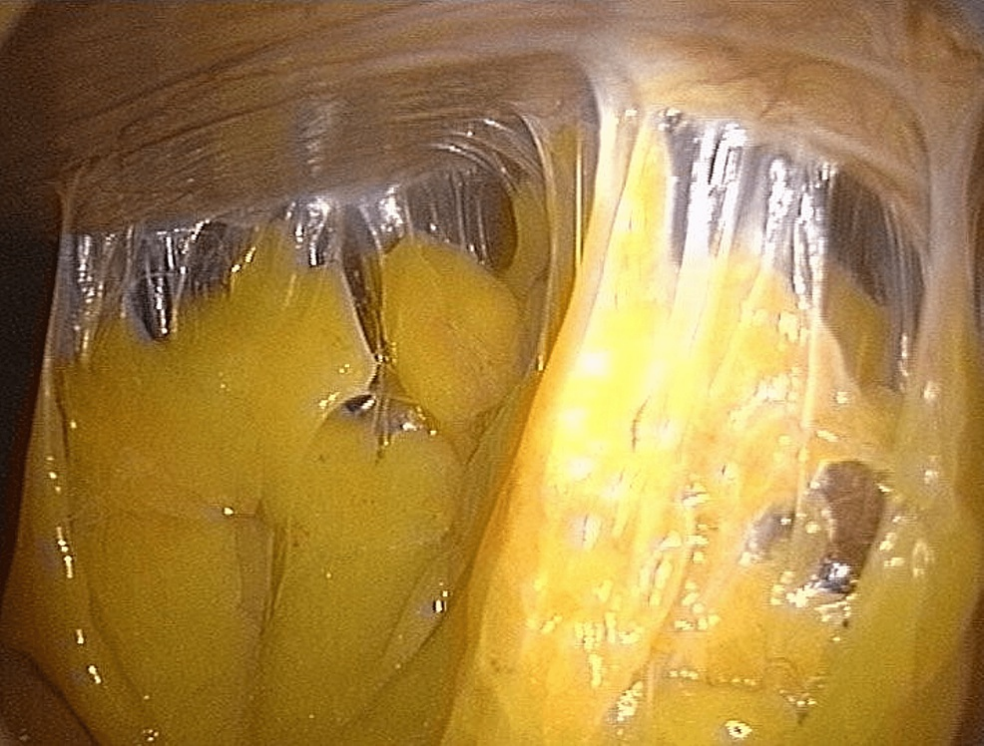
The image (from laparotomy) shows the presence of adhesion formations (translucent tissue) between the parietal peritoneum and the adipose tissue, in the absence of previous abdominal surgery. Image by Bordoni Bruno.

Chronic pain (persistence of pain beyond three months) in the presence of adhesions is able, like other chronic pathological situations, to alter the connectivity between areas of the central and peripheral nervous system [7]. This dysfunction leads to a dysregulation of the balance between the neurotransmitter gamma-aminobutyric acid (GABA) (inhibitory function) and glutamate (excitatory function and GABA precursor): this has only been studied in adolescents [8]. This imbalance prevents proper neuroplasticity, building persistent nociceptive patterns (central sensitization) [8]. Another contributing cause of chronic pain could be linked to an alteration of the adenosine triphosphate (ATP) receptor, in particular, P2X7; the latter is involved in the transmission of pain in the central nervous system (thalamocingulate circuits) [9]. Many brain areas are activated by a nociceptive stimulus, such as the somatosensory cortex (CSI, CSII), anterior cingulate cortex, putamen, posterior and anterior insular area, thalamus, and amygdala [10]. Nociceptive afferents pass first through the peripheral pathways. In chronic pain there is a spinal nociceptive hypersensitivity (SNH) [11]. Medullary laminae I, V, VI, VII, and X contain specialized nociceptive neurons and wide dynamic range (WDR) neurons; these neurons process the information deriving from distant anatomical areas and which, finally, they send to higher centers [10]. In the presence of SNH, not only nociceptive neurons are activated, but also WDRs, complicating the symptomatologic picture and causing phenomena of allodynia and hyperalgesia [10]. The recruitment of spinal neurons can be progressive or use the phenomenon of spatial summation [10]. According to some Authors, spinal glial cells play an important role in transforming medullary afferents into chronic nociceptive information, while glial cells located in the central nervous system play a role in negatively influencing the patient's psyche in the presence of nociceptive afferents [12]. Another mechanism that causes abdominal pain in the presence of adhesions is intestinal dysmotility caused by non-physiological tissue constraints [13]. This alteration of movement could generate intestinal dysbiosis [14]. Intestinal dysbiosis alters the function of the microbiota, which dysfunction alerts spinal and central glial cells, as well as astrocytes in the brain [15,16]. The intestine can produce different molecules (microbe-derived neurotransmitters) able to pass the various intestinal and peritoneal barriers and reach the medulla and/or the central nervous system (brain-intestine axis) [15]. These neurotransmitters stimulate glial cells, astrocytes (and oligodendrocytes) to produce pro-inflammatory substances and alter myelin, disturbing the correct neural transmission, and generating a pain and inflammation loop [15,16]. If the pain or dysfunction is chronic there will be a structural and functional change of the nervous system [17]. The clinician using manual medicine could approach the patient not only focusing on the area of adherence and possible source of pain and symptoms, but also on the possible neurological connections between the peritoneum and the central nervous system (Figure 1).

Osteopathic manual medicine and manual therapy

The literature offers few data derived from the application of manual therapy and only on an animal model; we have no real data on humans and the treatment of peritoneal adhesions [18]. The concept is to apply massages on the abdomen or stimulate intestinal mobility with the operator's hands to reduce the hypomobility of the tissues and avoid the friction of the different layers; these two events, if present, would stimulate the formation of adhesions, as detailed in the first part of our work [3]. With such approaches, the number of adhesions decreases compared to groups of untreated mice [18-20]. Manual therapy in these studies had a preventive objective. Applying this methodology of visceral mobilization to the patient in the presence of fresh sutures or areas of drainage is probably not feasible or would be risky. Furthermore, we do not know whether the peritoneal adhesions associated with the treatment that the mice underwent have a persistent result; knowing that a chronic inflammatory environment feeds the injured peritoneum (for years), we could assume that further adhesions may form [3]. In clinical routine, it is much easier to put one's knowledge into practice with patients with ascertained adhesions, suspected presence of peritoneal neoformations due to previous injuries or traumas, or inflammations/infections. In patients with confirmed presence of peritoneal adhesions, manual treatment should not have the aim of breaking the adhesions or creating micro-lesions, as not only is the inflammatory response further stimulated but, above all, the adhesions are not broken with manual medicine [3,21]. Manual medicine should implement very delicate techniques in the area of peritoneal adhesions in order not to stimulate the inflammatory response, in which the immune response is always strongly reactive to mechanical stress [3].

Possible symptomatologic pictures that go beyond the only symptom of pain

Osteopathic medicine looks not only at the symptom but also at the origin of the symptom; the symptom and its source do not necessarily coincide. Often, the patient presents to the clinician with a disorder whose origin he does not know. Observing only a targeted treatment exclusively where the pain occurs does not always resolve it or bring the maximum possible benefit. We know that nerve pathways transport multiple biochemical substances in an antidromic and orthodromic manner [22]. The presence of peritoneal adhesions can disturb intestinal function and create abnormal tensions in all tissues and structures that directly or indirectly involve these adhesions. In addition to nociceptors, other afferents (low-threshold sensitivity) can carry information related to pain as well as the molecules produced by the lesion/inflammation; these afferents are referred to as "wide-dynamic sensitivity" [23]. Each receptor can behave in autocrine and paracrine modes, influencing and being influenced by many neurotransmitters; the latter can retrace the path of the axon and involve the activity of different medullary neurons at multiple levels of the medullary axis [24]. Nociceptors and receptors capable of transmitting nociceptive information can alter (increasing or decreasing) the magnitude of the stimulus that will reach the central nervous system [24]. Furthermore, the same receptors can produce neurotransmitters (thanks to a self-generation of electric potential) capable of altering the tissue they innervate [24]. In general, viscera-derived afferents cluster in the spinal dorsal root ganglia (DRG), but the molecular information released by visceral receptors can arrive in any medullary area and/or involve the sympathetic and parasympathetic systems [22,25]. Furthermore, in the neurophysiological reality of the living, the information coming from the visceral area is indistinguishable from the somatic one at the medullary level (due to the synaptic convergence of the viscera-soma), making it difficult to schematize a symptom that necessarily derives from the viscera or from the somatic structure [10]. Not only that, but there is a macroscopic intercommunication of the rami communicantes involving the dorsal roots of adjacent spinal nerves; this organization concerns the entire path of the medullary axis, making a clinical decision on the origin of the symptom non-immediate [26,27]. Pain deriving from peritoneal adhesion (stimulated by mechanical and other events) does not necessarily take place in the area of origin; it does not necessarily express itself in a somatic area; and it can manifest itself in a psychological (anxiety, depression) and cognitive (altered concentration) sphere. "While nociception represents activity in specific sensory neurons, pain is more complex. Pain is a subjectively perceived experience that is rooted in a distributed and extensive network of neurophysiological interactions" [28]. The distribution of visceral innervation at the medullary level is not yet clear. For example, the vagal ramifications carry a specific visceral stimulus and not what concerns the whole bowel; this means that the visceral metamere does not necessarily coincide with the anatomical location of the viscera [29]. We know that a problem in the peritoneum that covers the gallbladder can alter the behavior of the shoulder (in general, of the right shoulder); this area is innervated by the phrenic nerve, which can carry altered mechanical information (not necessarily related to pain) towards the anastomotic network created by the same phrenic nerve [22,30]. The symptom will be expressed where, probably, the information arrival area is less capable of withstanding chronic electrical/biochemical stimuli. We know that a substance produced by peritoneal nociceptors and capable of traveling throughout the body, such as calcitonin gene-related peptide (CGRP, vasodilatory neuropeptide), can be found in the synovial fluids of the knee (animal model) [31]. We know that an excess of CGRP in the knee joint can lead to the onset of osteoarthritis [32]. We can hypothesize that a chronic knee disorder could be caused by an excess of paracrine secretion of CGRP from the peritoneum in the presence of post-surgical adhesions. The receptors and the different afferents that derive from the peritoneum, both from the peritoneal and visceral layers, constitute, together with other bodily afferents, the proprioceptive system [33]. The peritoneum is under constant stress from breathing, changes in blood and lymphatic flows, and movements of the trunk and abdomen. [34]. The proprioceptive system influences cognitive, motor, and emotional behavior, passing through the nucleus of the solitary tract of the vagus nerve, the spinal trigeminal nucleus, towards the limbic area, areas of the somatosensory cortex, and the area of the visual cortex [35]. If proprioceptive information is altered, multiple disorders can occur over time, such as neuromotor incoordination (increased falls), cognitive decline (decreased memory), behavioral changes (depression, anxiety), and decreased pain tolerance [35-38]. These bodily alterations are accentuated if there is an inflammatory picture [39-41]. We can hypothesize that these symptoms, which are not easy to classify, could be connected to the presence of peritoneal adhesions. In evaluating the patient, it is always advisable to palpate the abdomen.

Manual treatment hypothesis

**Figure 2:**
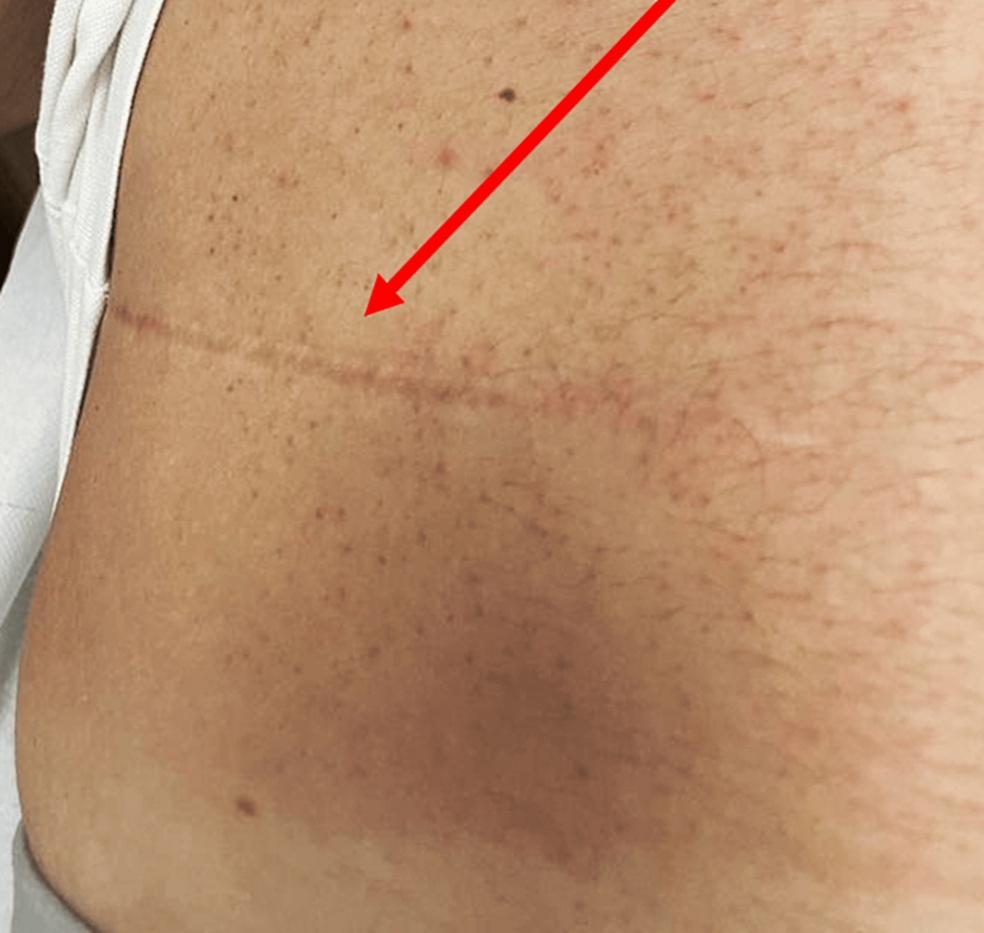
The image shows a horizontal scar (red arrow) on the right lower quadrant of the abdomen from previous ureteral surgery. On palpation, below the scar, the tissue appears dense and painful, with the probable presence of peritoneal adhesions. Image by Bruno Bordoni.

**Figure 3:**
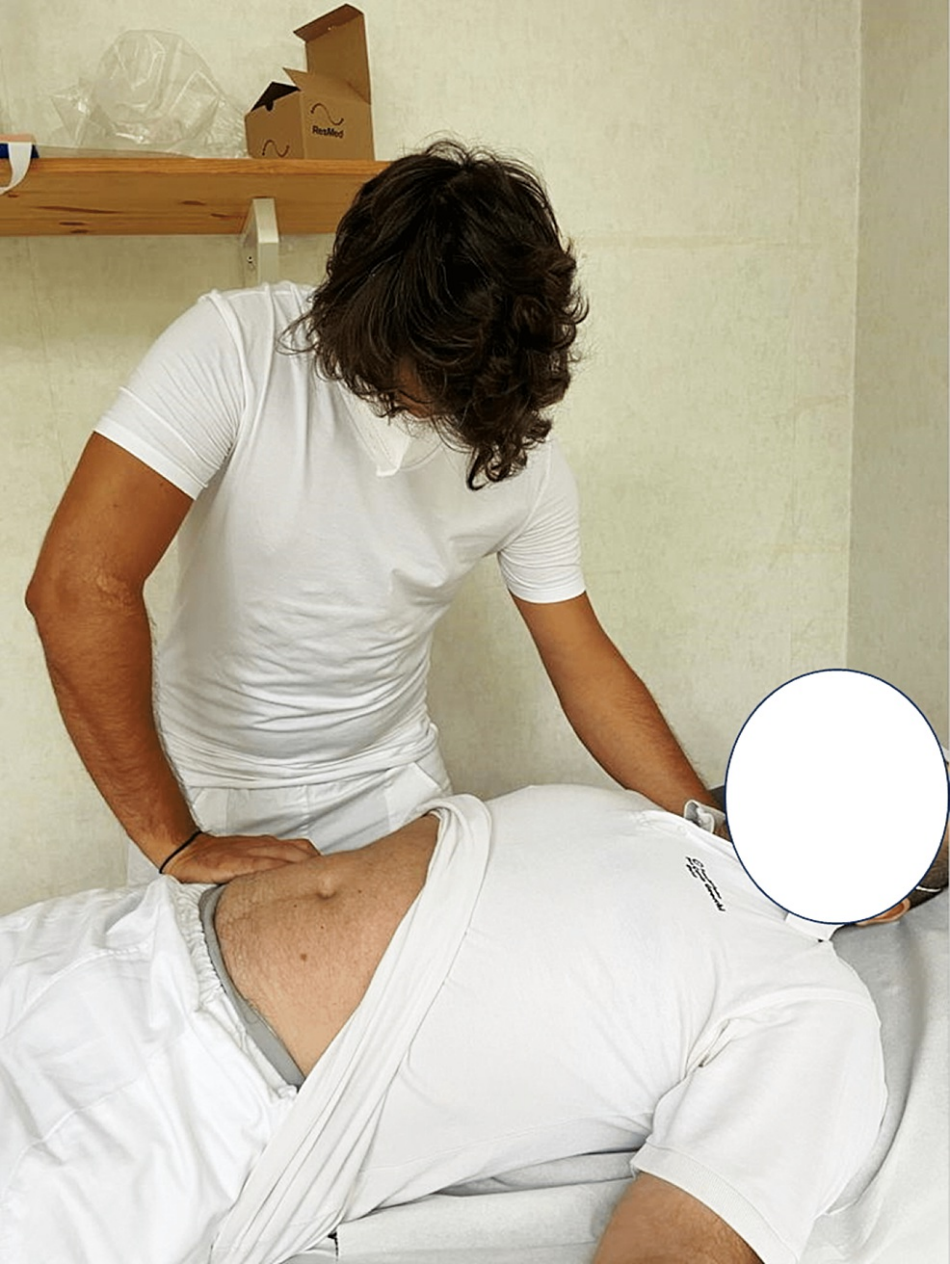
The image shows the operator with one hand on the area of possible adhesions and one hand under the cervical area. The technique applied is an indirect or listening technique, without inducing any movement; the technique is finished when the palpated tissues have a significant decrease in the tension felt (the tissues "melt"). Image by Bruno Bordoni.

A treatment that we propose and that comes very close to the work of Johnston is the palpatory agreement between the area of peritoneal adhesion (ascertained by history and palpation) and the zone of greatest stiffness of the paraspinal musculature determined by palpation. Johnston devised an indirect osteopathic approach, where it is necessary to find a tensional balance of the tissue (muscles, joints), seeking this balance through local or distant movements of the column and/or using the limbs [42,43]. To give an idea of the concept, it is like wanting to bring a center of mechanical tension back to its original position (where all the movement vectors intersect), which center, in the presence of dysfunction, was not at the right point. For further clarification, we recommend reading the volume by the same author [44]. Taking a cue from this approach, one hand of the operator is on the area of the adhesion (sore and stiff tissue), pressing up to the tissue corresponding to the adhesion, while the cranial hand is placed on the more rigid paraspinal area previously palpated (and often sore) (Figures 2-3). The patient is supine, and the operator is at his side. The technique can take several minutes and ends only when the palpatory perception of the clinician has highlighted the disappearance of tissue tension (like melting ice cream) in the treated areas. Furthermore, on re-palpation of the affected areas, the pain should be reduced or disappear. The duration of the effect will depend on the skill of the operator and the extent and chronicity of the adhesion. The clinician does not induce anything but waits for the tissues to respond autonomously, as one of the pillars of osteopathic philosophy teaches [45]. The possible reasons for this phenomenon have been discussed in a previous article [46]. We can define it as a listening technique.

**Figure 4:**
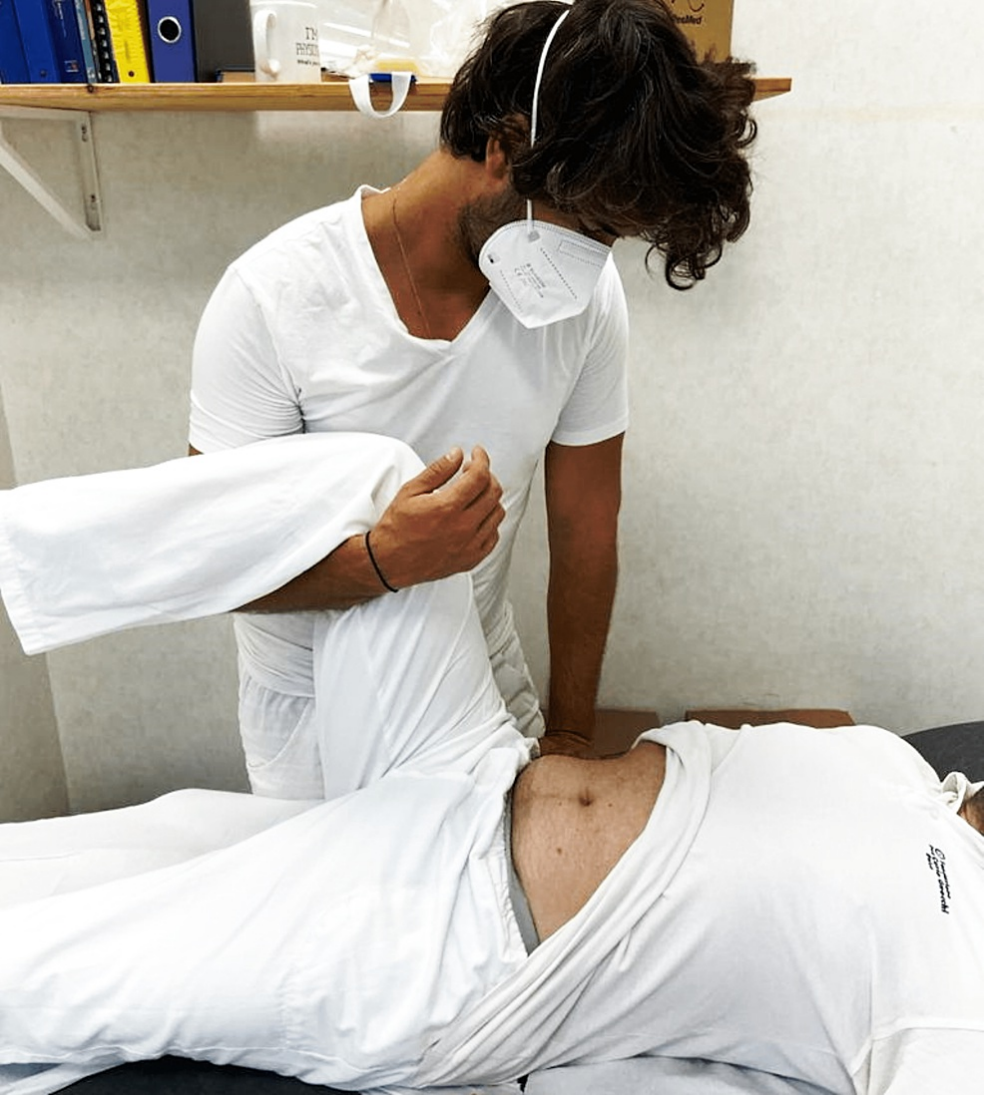
The image shows the operator placing a hand on the site of possible adhesion, and with the other hand holding the lower limb (in this case ipsilaterally) in the most optimal position to warn that the abdominal area under palpation is comfortable or with "zero" tension. The technique ends when the tissue under the palm of the hand is "dissolved." Image by Bruno Bordoni.

Another approach that can be performed in the presence of peritoneal adhesion is the use of the lower limb. The operator places one hand listening to the area of possible adhesion (pressing until the tissue corresponds to the adhesion), while with the other he holds the leg (or rests it on his own body) (Figure 4). With this approach, the hand on the abdomen listens, not inducing any movement, while the caudal hand tries to move the lower limb (or both limbs) to a position where the tension felt by the cranial hand drops to "zero." The clinician remains in the position that allows the tension in the abdominal area to be considerably reduced until he perceives that the tissue under the palm of the hand is "dissolved." The concept is to put the peritoneal tissues at ease until creating a mechano-metabolic environment capable of modulating the afferent response, which can stimulate more physiological afferents [46].

To conclude, let us recall the meaning of evidence-based medicine (EBM), since positing hypotheses of manual treatment does not represent an act outside scientific medicine: "..the EBM is based on equating the importance of existing literature with the experience of the clinician and the patient's experience with respect to the care received..." [47]. EBM is a triad made up of scientific knowledge, the experience of the clinician, and the experience to which the patient has been exposed after treatment. Thinking that using only current knowledge allows clinical progress becomes an obstacle to finding the most suitable strategy for the patient's health [47]. The undisputed evidence is that there is a lack of information on the causes that lead to the formation of peritoneal adhesions and that we have few shareable data points on the effects that manual treatment can have on the patient.

## Conclusions

Peritoneal adhesions are the local and systemic response to a lesion due to different causes, such as surgery, the presence of inflammation or infection, or abdominal trauma. Generally, the most evident symptoms are connected to localized pain and visceral intestinal disorders. The adhesions can cause other symptoms that are not so easily or immediately related to their formation. The task of every clinician is to make a correct diagnosis, which can be carried out with instruments or a thorough medical history and abdominal palpation. Osteopathic medicine looks not only at the symptom but also at the origin of the symptom; the symptom and its source do not necessarily coincide. There is no manual treatment that is considered the gold standard for symptomatologic improvement, but we can hypothesize that a gentle approach is the most suitable one, considering the current scientific knowledge we have on the processes of peritoneal adhesion formation. Further efforts must be made to identify the best osteopathic strategy for the well-being of the patient.
